# CD23-positive, *BCL2*-Rearrangement-negative Keimzentrumslymphome

**DOI:** 10.1007/s00292-023-01250-0

**Published:** 2023-11-06

**Authors:** Thomas Menter, Leticia Quintanilla-Martinez

**Affiliations:** 1 Pathologie, Institut für Medizinische Genetik und Pathologie, Universitätsspital Basel, Universität Basel, Schönbeinstr. 40, 4031 Basel, Schweiz; 2 Institut für Pathologie und Neuropathologie und Comprehensive Cancer Center Tübingen, Universitätskrankenhaus Tübingen, Eberhard-Karls-Universität Tübingen, Tübingen, Deutschland

**Keywords:** Follikuläres Lymphom, SOCS1, STAT6, ICC-Klassifikation, WHO-Klassifikation, Follicular lymphoma, SOCS1, STAT6, ICC classification, WHO classification

## Abstract

Im Rahmen der Erkenntnis, dass die Gruppe der follikulären Lymphome als sehr heterogen anzusehen ist, wurde in den letzten Jahren eine Gruppe follikulärer Lymphome abgegrenzt, die sich durch ein oft diffuses Wachstum (ohne Ausbildung follikulärer Strukturen) sowie Expression von CD23 in den Lymphomzellen und das Fehlen der klassischen *BCL2*-Translokation auszeichnete. Weitere Merkmale sind eine bevorzugte inguinale Lokalisation der Lymphome sowie ein lokalisiertes Stadium mit einer guten Prognose. Genetisch zeichnet sich diese Lymphomgruppe durch eine hohe Rate an *STAT6*- beziehungsweise *SOCS1*-Mutationen aus.

Die ICC-Klassifikation trug dieser Entwicklung durch die Einführung der provisorischen Entität „CD23-positives, *BCL2*-Rearrangement-negatives Keimzentrumslymphom“ Rechnung. Weitere Studien müssen nun zeigen, wie genau sich diese Entität definieren lässt (Kombination aus Morphologie, immunhistochemischem Phänotyp, Fokus auf genetische Alterationen), um so den Weg hin zu einer einheitlichen Klassifikation und auch einer besseren klinischen Charakterisierung dieser Fälle – insbesondere auch im Hinblick auf die möglichen therapeutischen Optionen – zu ermöglichen.

## Abstract (en)

Acknowledgeing that the group of follicular lymphomas is to be regarded as very heterogeneous, a group of follicular lymphomas has been delineated in recent years that was characterised by an often diffuse growth (without formation of evident follicular structures) as well as expression of CD23 in the lymphoma cells and the absence of the classic *BCL2* translocation. Further characteristics are a preferred inguinal localisation of the lymphomas and a localised stage with a good prognosis. Genetically, this lymphoma group is characterised by a high rate of either *STAT6* or *SOCS1* mutations.

The ICC classification took this development into account by introducing the provisional entity CD23 positive, *BCL2* rearrangement-negative germinal centre lymphoma. Further studies must now show how exactly this entity can be defined (combination of morphology, immunohistochemical phenotype, focus on genetic alterations) in order to pave the way towards a uniform classification and a better clinical characterisation of these cases – especially with regard to possible new therapeutic treatment options.

Zahlreiche Studien der letzten Jahre haben gezeigt, dass die Gruppe der follikulären Lymphome sehr heterogen ist. Neben dem „klassischen“ follikulären Lymphom, definiert durch seine Morphologie mit starren neoplastischen Follikeln, einer Koexpression von CD10 und BCL2 sowie dem Nachweis einer *BCL2*-Translokation, die ca. 80 % der follikulären Lymphome ausmachen [3], lassen sich hier die gut belegten und anerkannten Gruppen der *in situ* follikulären Neoplasie, des duodenalen follikulären Lymphoms, des testikulären follikulären Lymphoms, des kutanen Keimzentrumlymphoms, des pädiatrischen follikulären Lymphoms und des großzelligen follikulären Lymphoms mit *IRF4*-Rearrangement abgrenzen [1, 2]. Bis auf die *in situ* follikuläre Neoplasie und das duodenale follikuläre Lymphom zeigen diese Entitäten klassischerweise kein *BCL2*-Rearrangement. Eine weitere Entität des follikulären Lymphoms, das CD23-positive *BCL2*-Rearrangement-negative Keimzentrumslymphom, wird in diesem Beitrag diskutiert und vorgestellt.

## Der Weg von der Erstbeschreibung zur provisorischen Entität in der ICC-Klassifikation

**Figure 1:**
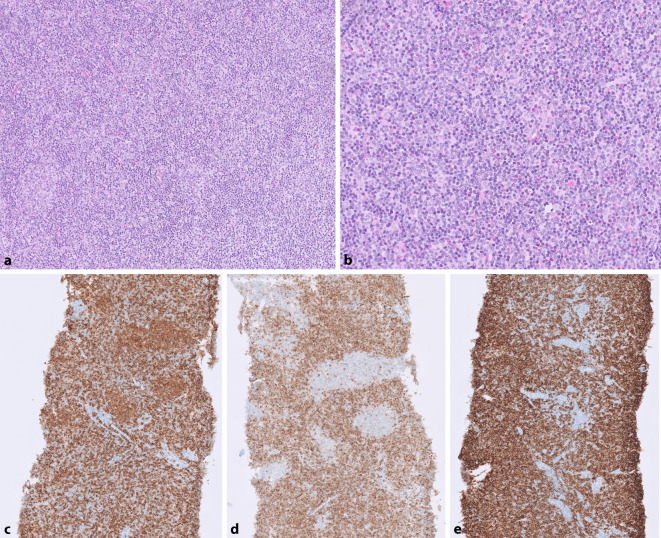

Bereits 2009 wurde eine weitere Subgruppe der follikulären Lymphome postuliert, die sich primär durch ein diffuses Wachstumsmuster mit Zerstörung der normalen Lymphknotenarchitektur auszeichnet [5] (siehe Abb. 1). Diese Fälle fanden sich primär in inguinaler Lokalisation, zeigten eine diffuse Positivität für CD23 und kein *BCL2-*Rearrangement. Ein weiteres morphologisches Charakteristikum ist der Nachweis sog. Mikrofollikel, was oft erst in der immunhistochemischen Färbung für B‑Zell-Marker deutlich wird. Es konnte gezeigt werden, dass es sich bei den Mikrofollikeln um Anteile des Lymphoms und nicht um bestehende reaktive Follikel handelt (siehe Abb. 1c, d). Auch Netzwerke der follikulären dendritischen Zellen sind hier nicht ausgebildet [11]. Aufgrund der fehlenden *BCL2*-Translokation sind die Lymphomzellen nur sehr schwach positiv oder meist komplett negativ für BCL2. Andere klassische Keimzentrumsmarker (GCET, HGAL, LMO2, BCL6, MEF2B) sind hingegen exprimiert. In der CD23-Färbung zeigt sich neben der schon erwähnten Positivität der Lymphomzellen kein Netzwerk follikulärer dendritischer Zellen.

Weitere Studien in den folgenden Jahren bestätigten das Vorhandensein dieser Fälle und konnten auch verschiedene genetische Charakteristika dieser Lymphomgruppe herausarbeiten, auf die im Folgenden noch detailliert eingegangen wird [9, 11, 12].

Diese Befunde führten dazu, dass die ICC-Klassifikation diesen Subtyp des follikulären Lymphoms als provisorische Entität aufnahm („CD23-positives, *BCL2*-Rearrangement-negatives Keimzentrumslymphom“) [2]. In der neuen WHO-Klassifikation findet sich hierfür keine genaue Entsprechung, die meisten dieser Fälle werden hier in der Gruppe der klassischen follikulären Lymphome mit ungewöhnlichen Merkmalen subsumiert [1].

## Einblicke in die Genetik

Schon die erste Studie aus dem Jahr 2009 konnte genetische Charakteristika – abgesehen vom Fehlen der *BCL2*-Translokation – herausarbeiten. So beschrieben Katzenberger et al. einen Verlust im 1p36-Locus in 93 % ihrer Fälle [5]. In der Analyse weiterer größerer Kohorten wurde diese Zahl deutlich nach unten korrigiert und liegt bei ca. einem Drittel der Fälle [6]. Eine weitere typische genetische Veränderung sind aktivierende *STAT6*-Mutationen. Die STAT6-Aktivierung führt zu einer Aktivierung des Interleukin-4(IL4)-Signalwegs, was letztendlich auch zur Überexpression von CD23 führt. Die CD23-Expression kann so als Surrogatmarker für die *STAT6*-Mutation angesehen werden [4, 6]. Des Weiteren zeigte sich, dass in *STAT6*-negativen Fällen oft Mutationen in *SOCS1* gefunden werden, auch dieses Gen ist am IL4-Signalweg beteiligt [6].

Letztendlich konnten 2 genetische Subgruppen der *STAT6*-mutierten follikulären Lymphome definiert werden [6]: Die eine Gruppe ist charakterisiert durch Mutationen oder ein „loss of heterocygosity“ (LOH) von *TNFRS14*, das auf dem schon erwähnten 1p36-Locus liegt, sowie Mutationen in *CREBBP* und *EZH2*. Diese Gruppe zeigt große Überlappungen mit den klassischen follikulären Lymphomen. Die zweite Gruppe zeigt auch *CREBBP*-Mutationen, jedoch keine Mutationen in *EZH2* und *TNFRS14*.

## Sind alle CD23-positiven follikulären Lymphome gleich?

Durch die mittlerweile vorhandenen größeren Kollektive an Fällen, die gemäß den vorgegebenen Merkmalen (CD23-Positivität, kein *BCL2*-Rearrangement) in diese Kategorie passen, zeigt sich jedoch eine gewisse Heterogenität. So weisen bei weitem nicht alle Fälle ein diffuses Wachstumsmuster auf (in manchen Kohorten nur bis zu 30 %). Auch teilweises oder vollständiges follikuläres Wachstum wie beim klassischen follikulären Lymphom sind dokumentiert [6, 9, 11]. Zudem treten die Lymphome nicht nur inguinal auf. Auf der anderen Seite ist jedoch die Kombination aus diffusem Wachstum und inguinaler Lokalisation sowie niedrigem klinischem Stadium sehr typisch.

Um die Rolle der CD23-Expression besser zu verstehen, haben wir eine größere Kohorte von follikulären Lymphomen mit Selektion auf CD23-Expression unabhängig von anderen Parametern untersucht. Erste Analysen zeigten, dass hierbei unterschieden werden muss, ob eine diffuse Expression von CD23 vorliegt oder nur eine Expression von CD23 in den Lymphomzellen in der interfollikulären Zone. Lediglich die erste Gruppe zeigt auch eine Prädominanz von *STAT6*/*SOCS1*-Mutationen. Bei der Expression von CD23 in der interfollikulären Zone liegt dies meist an Interaktionen mit dem Tumor-Microenvironment und zeigt keine Assoziation mit *STAT6/SOCS1*-Alterationen [7]. Derzeit führen wir noch Genexpressionsstudien durch, um weitere Einblicke in die Pathogenese dieser Lymphome zu erlangen. Bekannt ist diesbezüglich schon, dass auch das Tumor-Microenvironment eine große Rolle spielt, da auch follikuläre T‑Helferzellen IL4 produzieren können, was zur Proliferation und zum Überleben der Lymphomzellen beitragen kann [7].

Mit der Positivität für CD23 kommen auch potenzielle andere Lymphomentitäten in die Differentialdiagnose, die aber gut morphologisch beziehungsweise durch weitere immunhistochemische Untersuchungen ausgeschlossen werden können: Die CLL/SLL zeigt neben der CD23-Expression auch eine Positivität für CD5 und LEF1. Das Mantelzelllymphom und das Marginalzonenlymphom können gemäß Literaturangaben in seltenen Fällen eine schwache CD23-Expression zeigen [8, 10]. Die Diagnose des Mantelzelllymphoms kann durch seinen spezifischen Phänotyp (Koexpression von CD5, Cyclin D1 und SOX11 in den nichtleukämischen Fällen) leicht gesichert werden. Beim Marginalzonenlymphom ist dies schwierig, da hier spezifische immunhistochemische Marker fehlen, morphologische Hinweise wären eine Kolonisierung bestehender reaktiver Follikel oder eine deutliche plasmozytoide Ausdifferenzierung sowie eine Leichtkettenrestriktion der Plasmazellen. Es gibt jedoch Anzeichen dafür, dass die beschriebenen CD23-positiven Marginalzonenlymphome doch eher der Gruppe der CD23-positiven Keimzentrumslymphome ohne *BCL2*-Rearrangement zuzuordnen sind (auch hier Nachweis von *STAT6*-Mutationen) [6].

## Ausblick

Die hier dargestellten Erläuterungen zeigen, dass eine weitere Untersuchung dieses Lymphomtyps und dessen Abgrenzungen von anderen Subgruppen des follikulären Lymphoms weiter voranzutreiben sind. Das Gefäß der „provisorischen Entität“ bietet hierbei die Möglichkeit, Patholog:innen auf diesen speziellen Subtyp aufmerksam zu machen und somit das Erkennen und die bessere Erforschung dieser Fälle zu erleichtern. Zudem muss bei einer entsprechenden Reevaluation dieser Gruppe auch kritisch die Definition der Grundkriterien (Morphologie, immunhistochemischer beziehungsweise genetischer Phänotyp) hinterfragt werden. Nur so kann es letztendlich gelingen, diese Lymphomgruppe klar abzugrenzen und so auch für unsere klinischen Kolleg:innen erkennbar zu machen. Dies sollte ja letztendlich das Ziel jeder Klassifikation sein, da so gezielte klinische Studien dieser „Subentität“ möglich sind, um die Therapie und Prognose der Patient:innen zu optimieren.

## Fazit für die Praxis

- Die Abgrenzung CD23-positiver Keimzentrumslymphome ohne *BCL2*-Rearrangement ist ein wichtiger Schritt zum besseren Verständnis dieser Entität.
- Der alleinige Fokus auf die CD23-Expression kann als unvollständig angesehen werden, jedoch zeigen insbesondere die *BCL‑2*-Rearrangement-negative Fälle mit diffuser CD23-Expression auch in den Keimzentren eine hohe Rate von *STAT6*/*SOCS1*-Mutationen.
- Die genaue Definition dieses Subtyps (basierend auf Morphologie, immunhistochemischem Phänotyp oder genetischen Merkmalen) ist noch nicht abgeschlossen und bedarf weiterer Studien an größeren Kollektiven.

## References

[1] Alaggio R, Amador C, Anagnostopoulos I, Attygalle AD, Araujo IBO, Berti E (2022). The 5th edition of the world health organization classification of haematolymphoid tumours: lymphoid neoplasms. Leukemia.

[2] Campo E, Jaffe ES, Cook JR, Quintanilla-Martinez L, Swerdlow SH, Anderson KC (2022). The international consensus classification of mature lymphoid neoplasms: a report from the clinical advisory committee. Blood.

[3] Carbone A, Roulland S, Gloghini A, Younes A, von Keudell G, Lopez-Guillermo A (2019). Follicular lymphoma. Nat Rev Dis Primers.

[4] Crouch S, Painter D, Barrans SL, Roman E, Beer PA, Cooke SL (2022). Molecular subclusters of follicular lymphoma: a report from the united kingdom’s haematological malignancy research network. Blood Adv.

[5] Katzenberger T, Kalla J, Leich E, Stocklein H, Hartmann E, Barnickel S (2009). A distinctive subtype of t(14;18)-negative nodal follicular non-Hodgkin lymphoma characterized by a predominantly diffuse growth pattern and deletions in the chromosomal region 1p36. Blood.

[6] Nann D, Ramis-Zaldivar JE, Muller I, Gonzalez-Farre B, Schmidt J, Egan C (2020). Follicular lymphoma t(14;18)-negative is genetically a heterogeneous disease. Blood Adv.

[7] Pangault C, Ame-Thomas P, Ruminy P, Rossille D, Caron G, Baia M (2010). Follicular lymphoma cell niche: identification of a preeminent IL-4-dependent T(FH)-B cell axis. Leukemia.

[8] Saksena A, Yin CC, Xu J, Li J, Zhou J, Wang SA (2019). CD23 expression in mantle cell lymphoma is associated with CD200 expression, leukemic non-nodal form, and a better prognosis. Hum Pathol.

[9] Siddiqi IN, Friedman J, Barry-Holson KQ, Ma C, Thodima V, Kang I (2016). Characterization of a variant of t(14;18) negative nodal diffuse follicular lymphoma with CD23 expression, 1p36/TNFRSF14 abnormalities, and STAT6 mutations. Mod Pathol.

[10] van den Brand M, van Krieken JH (2013). Recognizing nodal marginal zone lymphoma: recent advances and pitfalls. A systematic review. Haematologica.

[11] Xian RR, Xie Y, Haley LM, Yonescu R, Pallavajjala A, Pittaluga S (2020). CREBBP and STAT6 co-mutation and 16p13 and 1p36 loss define the t(14;18)-negative diffuse variant of follicular lymphoma. Blood Cancer J.

[12] Zamo A, Pischimarov J, Horn H, Ott G, Rosenwald A, Leich E (2018). The exomic landscape of t(14;18)-negative diffuse follicular lymphoma with 1p36 deletion. Br J Haematol.

